# The effect of glutamate-gated chloride current on the excitability of a Purkinje cell: a modeling study

**DOI:** 10.1186/1471-2202-12-S1-P154

**Published:** 2011-07-18

**Authors:** Shiwei Huang, Erik De Schutter

**Affiliations:** 1 Computational Neuroscience Unit, Okinawa Institute of Science and Technology, Okinawa 904-0411, Japan; 2 Theoretical Neurobiology, University of Antwerp, B-2610 Antwerpen, Belgium

## 

Purkinje neurons express, in high abundance, a glutamate gated chloride channel commonly known as the Excitatory Amino Acid Transporter subtype 4 (EAAT4). EAAT4 belongs to the family of glutamate transporters, which in mammalian nervous system is responsible for clearing synaptic glutamate [1]. Studies of these transporters in heterogeneous expression systems demonstrated that in addition to glutamate transport, the binding of glutamate to the transporter activates a chloride current through the transporter with properties liken that of a channel and which is functionally independent from the transport process [2].

The role of the chloride channel in glutamate transporters is only known for the EAAT subtype 5 (EAAT5). On rod bipolar cell axon terminals, EAAT5 activation by glutamate results in membrane hyperpolarization, which consequently inhibits terminal glutamate release [3]. Whether the chloride channel of EAAT4 has a physiological role in Purkinje neurons remains unknown. A synaptic model was developed to determine conditions in which the chloride channel of EAAT4 could influence Purkinje neuron function and whether these conditions are physiologically relevant.

The model comprises a single compartment with uniform distribution of AMPA receptors and EAAT4. The EAAT4 model is based on a 16-state kinetic model of EAAT2 [4] using reaction rates of EAAT4 measured in [5]. AMPA receptor conductance and absolute permeability of EAAT4 were parameterized for adjusting their respective current amplitudes. The model reproduced the EPSC and EAAT4 channel currents evoked in a Purkinje neuron by parallel fiber stimulation under conditions similar to those used in [5]. The result serves as a basis for investigating the effects of the EAAT4 chloride current on Purkinje neuron excitability and intracellular chloride concentration.
